# Supplementary material for: Clinical ethics consultation among Italian ethics committee: A mixed method study
Source: PLoS One. 2019 Dec 30;14(12):e0226710. doi: 10.1371/journal.pone.0226710 (PMC6936824; doi:10.1371/journal.pone.0226710)
Supplement: Attachment — Submitted filename: Response letter_ 25-07-2019.docx [file pone.0226710.s001.docx]

Title: **“RESEARCH ETHICS CONSULTATION AND CLINICAL ETHICS CONSULTATION: A MIXED METHOD STUDY”**

Dear Editor,

Please find enclosed the article: **“RESEARCH ETHICS CONSULTATION AND CLINICAL ETHICS CONSULTATION: A MIXED METHOD STUDY”** that we are submitting in a new version for publication in “Plos One”.

In the first submission we received several critiques and suggestions that we considered as properly addressable to significantly improve the manuscript.

We contacted the Editor to see whether PlosOne would eventually consider a new submission of the revised manuscript, and the Editor granted our request, as explained in the cover letter.

This is the rebuttal letter including our point-by-point response to the original reviewers’ concerns.

We would like to thank the Editor and the reviewers for their constructive reviews.

| **Editor’s and Reviewers’ comments** | **Response to Editor and Reviewers** |
| --- | --- |
| Thank you for submitting this manuscript on a worthwhile topic. Both reviewers have provided thorough reviews with the second reviewer also marking edits on the manuscript itself which I think is very helpful. While there is little research on RECs, the current state of the manuscript and presentation of the results require substantive rewriting and at this stage I would recommend rejecting the manuscript.  Both reviewers echo a couple of major concerns which I also share. | We agreed with all the points raised and conclude that critiques and suggestions can be properly addressed and  would make a significant improvement to the manuscript. |
| First, the definition of what is included as a REC is ill defined and unclear. | We redefined EC, REC and CEC . |
| Second, there seems to be a lack of methodological clarity on what exactly was done in terms of the questionnaire and interviews. | We modified the manuscript (Materials and Methods) to make the methodological aspects clear; in the quantitative phase of the study questionnaires were administered to ECs secretariats to collect data on ethics consultation activity; in the qualitative phase, a semi-structured interview was administered to selected key-informants with the aim of further exploring the information gathered from the quantitative phase on significant elements of ethics consultation and to probe significant survey’s results. |
| There is little description in terms of how the questionnaire was developed, validated/pretested and implemented. | The questionnaire was very simple and included 3 thematic areas. It was developed by the research team to collect specific data to address the survey’s basic research questions, as explained in the Data collection and analysis section. |
| The type of analysis (descriptive statistics) used and why. | As far quantitative data the analysis was merely descriptive; we reported % among all EC by geographical regions and within grouped regions. We do not think is necessary to state that these % are descriptive statistics given that they are indicated in the Tables |
| The qualitative methods and analysis is also poorly described.  And both the instrument and interview guide should be made available. | We expanded the description of the qualitative methods and analysis. We included the interview guide. |
| Lastly, I share a similar concern again by both reviewers in that the presentation and writing needs improvement. | We thoroughly revised the manuscript and we seek for the help of an English mother tongue (acknowledged). |
| Reviewer 1:   1.1 Dear Authors - Your use of REC is a bit confusing. Sometimes it seems to refer to research ethics committee (like an IRB in the use) sometimes they seem to be using it as a blanket term for ethics committee (research and clinical), and sometimes it seems to mean research ethics consultation. As a reviewer it wasn't clear to me whether you were studying ethics committees, clinical ethics committees, or research ethics committees (in the use called IRBs) or all of the above. | Thanks for the comment, it helps in definition of terms. We clarify the use of EC, REC and CEC therminology. |
| 1.2 Your use of the term regulations in the context of RECs is not quite correct. I believe you want to use the words policies and processes, in that I believe you reviewed documents describing how the bodies operated, how they function, how they run themselves. | Yes, we replaced the term regulation with policy, as suggested. |
| 1.3 My understanding is that you wanted to see whether these committees actually provided consultative services. I think what would be helpful is if you defined what you mean by consultative services. Also please realize that in the use research ethics committee is the IRB (institutional review board) and the RECS is a research ethics consultative service, thus your use of the acronyms REC and RECs is a bit confusing to some readers. | Correct. We revised the manuscript and changed ECs definitions to make them clear to readers. |
| 1.4 Also some of the presentation of the data within the text doesn't add-up (lines 192 thru lines 214 -- 46 reported consult requests but it seems that 48 different commitees (again not certain if these are ethics committees, clinical ethics committees, or research ethics committees). | We revised the section according to this suggestion. |
| 1.5 See also the next paragraph: it is clear where the six RECs reported in 219-220 come from (part of the 23?). I would also highly recommend better descriptions for tables 2 and 3 --- it took this reviewer a bit of time to figure out what each was presenting. WHen I say better descriptions -- - this can be in the text as well as in labeling the tables and in a table legend. | Thank you for the suggestions, we modified tables and added a description. |
| 1.6 I would encourage you to have a colleague who is a native English speaker work with you on the writing of the manuscript Some of the words and phrasing you use is awkward (e.g."we phoned to the RECs who did not respond to the email asking the questions of the questionnaire through a telephonic interview"; i would suggest a re-write we phoned ethics committees that did not respond to the email reminding, collecting questionnaire response from them orally, of something like that. Telephonic is used in appropriately). | We revised the manuscript with an English mother tongue . |
| 1.7 Your N for the qualitative piece of the study is quite low and it isn't clear to me a) why and b) what types of solid conclusions you can make from those data. | Thank you for your comment. We are aware that the number of participants is limited.  Indeed, we planned to interview 8 key-informants with no replacement in case of refusal. Only 4 accepted to participate. Due to this reason, we were able to make solid conclusions from our data only for the two themes described in the article. |
| 1.8 Finally, I suggest you conduct a more thorough literature review... IRBs weren't formally established in the US until the mid-1970s as a result of the Belmont Report... You state RECs in the US were established in the 1960s. If you are referring to the formal, codified IRB structure, the correct time period is the mid-1970s; and i believe you will find a richer literature on teh study of both clinical ethics consultation services and research ethics consultation services which you should consider citing. | We improve the literature review as suggested. |
| 2.1 This study is an important contribution to international awareness of the practice of research ethics consultation.  For the article to be useful to readers outside Italy, the manuscript needs to present a fuller description of the study itself and of the context of Italian hospitals/clinical centers and the system of research ethics committees. | Many thanks. On the basis of these suggestions and comments, we improved the manuscript as requested. |
| 2.2 It also needs revision for English syntax and choice of words, primarily to replace false cognates between Italian and English and to clarify concepts that are expressed differently in idiomatic English than what would be the simple translation from the Italian | We revised the manuscript with an English mother tongue. |
| 2.3 First, for non-Italians, more information is needed to understand the context of the study and the scope of RECs' operations across the country.  It is not clear whether the hospitals surveyed are public or private, or how RECs are both established and governed in Italy.  Are hospitals and clinical centers the same thing? Are all hospitals (and/or clinical centers) goverened by national or regional laws that spell out specific standards on research and research review to ensure ethical protections of research participants? If there are private (e.g., religiously affiliated) hospitals that conduct research, are their research review processes and committees governed by internal rules as well as by governmental laws? | The revised manuscript addresses these concerns and we hope that the current version is clear |
| 2.4 With regard to the governance of RECs specifically, the manuscript uses the word "regulation(s)" exclusively, including to refer to the governing documents of the RECs in that the study collected and analyzed. In the US system, these documents would more likely be called "policies", especially if they were written for an individual alone by administrators inside the hospital's structure -- "regulation" would refer to external governance documents that have the force of law (local, regional, or national). | We replaced the term regulation with policy, as suggested. |
| Additionally, the manuscript describes the collection of "regulations"(policies) from the individual clinical centers' websites or secretariats. Are these documents expected to be publically available? | Yes, in Italy they are often publically available to meet the principle of transparency that is required for the Public Administration. |
| 2.5 The survey and subsequent interviews are described as surveys/interviews of RECs. Because different people on RECs may have quite different views and experiences of the same activity, the description of the survey should detail   - who the surveys were directed to (e.g., the head of the committee), - why individuals in that particular position were targeted, and - whether there were any safeguards to ensure that qualified individuals responded to the survey. | We added the requested information. |
| 2.6 In the survey or the interview, did you define "ethics consultation" as the basis for any of the questions?  If so, it would be helpful to the reader to include that definition in the manuscript's discussion of the responses. It would also be valuable to know whether any of the respondents gave a specific defintion of "ethics consultation".  In the US and Canada, research ethics consultation is a specialty service apart from the administrative ethical review of research protocols to ensure that they meet regulatory requirements. Here in the US, research ethics consultation is provided by individuals with advanced education and training in ethics and research.  This question is expecially relevant to the findings (lines 260-266) that health care professionals "often perform ethics consultation informally"... and that people think that they "could not benefit from specific training programs". It may be that there is typographical error or a gap in translation here, but the text currently suggests that respondents reported that health care professionals can do research ethics consultation without training (line 263). | No, we actually don’t define “ethics consultation” as the basis for the question, but a discussion on the definition emerged. Please, see the Discussion section.  We did not separated ethics consultantion in clinical and research since we investigated this matter being aware for cultural reasons there no such a clear distinction in Italy.  Perhaps our findings showed that many health professionals believe that ethics consultation can be provided according to personal sensibility even with a superficial knowledge of medical ethics, a belief that support the need for an appropriate training |
| 2.7 The activities described in the bullet-point list in lines 195-214, and the committee structures described in bulle- point list in line 228-241, seem disconnected from the broader context of the study and need additional introduction about whether these descriptions combine research and clinical ethics consultations or are just about clinical ethics consultation.  The order in which the topics in the bullet point at line 200 is presented seems chaotic -- which are clinical and which are research issues?  The observation in the bullet point in lies 214-215 needs to be presented in more detail - does this mean that 6 RECs do not keep records? | As resulted from our study quantitative and qualitative, the two forms of ethics consultation is indeed unclear.  We reviewed the bullet point. We hope now is clearer. |
| 2.8 Since PLoS ONE does not copy-edit manuscripts, it would be very helpful to have the manuscript reviewed and edited by someone who is a native speaker of English and bi-lingual in Italian. The text is generally readable, but there are several places in the manuscript where the languge is not clear. False cognates may also mislead readers. Committees are typically not referred to as "institutions", but rather as "bodies" usare bodies; "provide" means to give (in Table 3, "do" or "offer" would be better) whereas "provide for" means to "make available" or "ensure the availability of". And RECs are either "underappreciated" or "undervalued", not "underestimated". As a former editor of an international journal, I typically copyedit when I review manuscripts and I have included a scan of the manscript with my notes and queries in the hope that they may be helpful. | Many thanks for the scan of the manuscript, it was very helpful in revising our research.  We used all your suggestions, then we revised the manuscript with a native speaker of English and bi-lingual in Italian. |
| 2.9 I look forward to seeing a revised version of this manuscript published - it will be useful to many of us in the US who do both clinical and research ethics consultation. Thank you for the opportunity to review it. | Thanks for the comment. |
