# Supplementary material for: Clinical ethics consultation among Italian ethics committee: A mixed method study
Source: PLoS One. 2019 Dec 30;14(12):e0226710. doi: 10.1371/journal.pone.0226710 (PMC6936824; doi:10.1371/journal.pone.0226710)
Supplement: Attachment — Submitted filename: Response to reviewers.docx [file pone.0226710.s002.docx]

Ms. Ref. No.: PONE-D-19-21084

Title: **Clinical ethics consultation among Italian Ethics committee: a mixed method study**

Dear Editor,

Please find enclosed the article**: Clinical ethics consultation among Italian Ethics committee: a mixed method study** that we are resubmitting for publication in PLOS ONE after the invitation for a resubmission by the Editor.

Thank you very much for reviewing our manuscript. We also greatly appreciate the reviewers for their complimentary comments and suggestions.

Together with the other co-authors, we have discussed the comments you reported in your e-mail, and revised the article accordingly.

Please find attached a point-by-point response to reviewer’s concerns.

We hope that you find our responses satisfactory and that the manuscript is now acceptable for publication.

Journal requirements:

We note that you have included the phrase “data not shown” in your manuscript. Unfortunately, this does not meet our data sharing requirements. PLOS does not permit references to inaccessible data. We require that authors provide all relevant data within the paper, Supporting Information files, or in an acceptable, public repository.

*Sorry, it was a refusal. We added the open peer commentary published on the American journal of bioethics in the first submission.*

| **Editorial and reviewers’ comments** | **Response to editor and reviewers** |
| --- | --- |
| AE 1. The most significant revision required is that you clarify how your data suggests the "need to set up institutional bodies dedicated to ethical matters, composed by experts with a specific competence in ethical issues." Currently your data does not seem to show this. You need to more clearly establish a connection between your findings and this conclusion. | We think that the qualitative and quantitative results of our study show a lack of knowledge/information on ethics consultation, given the need to address the daily ethical issues that healthcare professionals face in clinical practice that are not related to research tasks. Moreover, the new EU regulation complicated this aspect. (Please, see the Conclusion Section). |
| AE 2. Spell out acronyms like REC and EC. -Can you clarify what this means? "Review of the policies promoted by ECs showed that 72,6 % (n=69) of all the ECs (N=95) being actually capable of providing ethics consultation service by policy." The meaning is unclear. What policy are you referring to? -Can you clarify this sentence? "To date, ethics consultation in clinical and research practice is largely underappreciated and NOT WELL? understood by users." It seems that you are arguing that it is underappreciated and it IS understood. I don't think this is what you mean. -This sentence does not make sense: "Despite research THAT? clinical ethics consultation services should not necessarily be mandatory, following the recent EU Regulation on clinical trials, institutional ethics consultation bodies should be re-evaluated. [END THE SENTENCE HERE.THE REST IS REDUNDANT] in perspective of application of new regulations." | -According to your suggestion, we spelled out the acronyms.  -We used policy as suggested by reviewers in the first review process (we used regulations in the first draft). Please, see line 42 and 48.  -Thanks for your comment. We rewrite the sentence in order to clarify its meaning. Please, see line 55,56  -We agree and corrected the sentence, please see line 58-60. |
| Ae 3. Please clarify why it is worth mentioning the Friuli Venezia Giulia Region in the reporting of the ECs with a policy on ethics consultation. | We modified the statement to clarify its meaning. Please, see line 246-47. |
| AE 4. Please rewrite this sentence on line 268: "The 23 ECs that did not perform ethics consultation by policy declared that they were supported by alternative structures, except for six ECs that did not provide ethic consultation and were not supported by any alternative body/structure." Do you mean 17 ECs that did not perform ethics consultation by policy declared that they were supported by alternative structures?? | Thanks for the comment, we corrected the sentence. Please, see line 270-272. |
| AE 5. The following section of the discussion is unclear: "Interestingly, the interviews and surveys led us to cover [NOT SURE WHAT YOU MEAN BY COVER] the definition of consultations despite THE FACT THAT? this was a direct question IN our survey or interviews. DEFINITIONS OF ETHICS CONSULTATION provided by participants, WERE in agreement with the Ministerial Decree of February 8, 2013 as any activity concerning ethical issues related to scientific and clinical activities, aiming at protecting and promoting a persons’ value (9)." It is not clear what you mean by including this section on the definition of ethics consultation. How is this related to the research question/ study design? | Thanks for your comment. First of all, we corrected the sencence following your suggestions (Please, see line 339-344).  We included this section because this was a specific request by reviewer n.2 in the first review process. |
| **Editorial and reviewers’ comments** | **Response to editor and reviewers** |
| R.1.1 I would consider the manuscript just sufficient and even if not completely well done it is acceptable for publication. About the survey through the use of questionnaires, few results are not so clear and precise like, as reported at Tab.2 (line 220), the definition of Clinical centers and the number of 6 for Ethics Committese in the Veneto Region (in fact in the Veneto Region the Ethics Committes are more than 6: the authors must clarify if they take in account only the RECs or also the CECs). | Thanks for the comments and suggestions.  About the Tab. n.2, we conducted a search of the 95 Italian ECs listed in the Italian Medicines Agency registry (AIFA) at December 2015, as explained at lines 169-171. In Italy there’s not an italian registry of CECs, but only RECs.  About the definition of clinical centers, they are clinical centers/clinical units that van perform research activities. |
| R.1.2 The number of interviews is very low and therefore it is difficult to have significant conclusions. | Thank you for your comment. We were aware that the number of participants was limited and agree that 4 interviews’ analysis can give a small understanding of the whole picture. Nonetheless, we restrict the analysis to what was recurrent through the interviews, trying to discuss the two emerging and transversal themes along with the results of the quantitative phase. |
| R.1.3 It should be better explained the use of the concept of the Clinical Ethics Consultation and in this context it is important to quote the recent Italian publications (as the Declaration of Trento). | Thanks for your comment. We agree about the importance to clarify the concept, but we chose to use only the MD definition, even if it is not the most exhaustive, with the aim to avoid confusion about the different meaning.  Our study is about the function of clinical ethics consultation in the italian ECs. |
| R.1.4 About the references, ref. nr. 3, line 384; ref. nr. 16, line 410-411, are not completed and quotations for the US Belmont Report and the Italian literature about clinical ethics consultation and ethics committees are missing. | We corrected references. |
| **Editorial and reviewers’ comments** | **Response to editor and reviewers** |
| R.2.1 To be of interest to a broader audience, it is suggested that the authors delineate the rationale for their work more clearly. They have assumed the reader has detailed knowledge of the EU regulations (this reviewer has read this particular EU regulation 536/2014) but does not draw any parallels between that regulation and the current submitted work. For example the justification for this work seems to be that the "its effect is far reaching and will likely effect aspects such as the role and function of ethics consultations". This is not sufficient most readers and certainly would be important to justify the current work to draw out the section in the current regulation that is pertinent. The impact, importance and why this work is of interest to a broader audience is lacking upfront. | Thanks for your suggestion, we understand this point of view.  In the background section, we used reference n.10 to deepen the analysis of the EU regulations and ref.n. 2 to explain why the italian NBC expressed concern about the function of handling ethics consultations after the EU regulations.  We added the “tasks beyond the evaluation of experimental study protocols”. |
| R.2.2 The survey methods, and survey itself and reporting are done are done with rigor and high quality. | Thanks for the comment. |
