# Supplementary material for: Clinical ethics consultation among Italian ethics committee: A mixed method study
Source: PLoS One. 2019 Dec 30;14(12):e0226710. doi: 10.1371/journal.pone.0226710 (PMC6936824; doi:10.1371/journal.pone.0226710)
Supplement: Attachment — Submitted filename: Response to reviewers.docx [file pone.0226710.s003.docx]

Ms. Ref. No.: PONE-D-19-21084R1

Title: **Clinical ethics consultation among Italian Ethics committee: a mixed method study**

Dear Editor,

Please find enclosed the article**: Clinical ethics consultation among Italian Ethics committee: a mixed method study** that we are resubmitting after revisions required.

Thank you very much for your comments and suggestions.

Together with the other co-authors, we have discussed the comments on the conclusion and revised it accordingly.

We hope that you find our response satisfactory.

| Additional Editor Comments |  |
| --- | --- |
| Unfortunately the revised paper does not address the central concern expressed in the most recent revision; that the data does not support the conclusion.  The data shows: -According to a review of policies, 72.6% of ECs provide ethics consultation (And 52.1% of them received no requests for ethics consultation in 2016). -According to the survey, 67.6% ECs provide ethics consultation. -Qualitative interviews with four people indicated that the team should be interdisciplinary, and that HPs perform ethics consultation informally. Further, "HPs could not benefit from specific training programs, and often lack information about the existing services and its activities."  I fail to see how this data supports the idea that there should be institutional bodies composed by experts who a specific competence in ethical issues. Who are these experts? Where have they been identified in the study?  The authors must significantly revise the conclusion in order to reflect the findings. As it stands, the data does not support “the need to set up institutional bodies dedicated to ethical matters, composed by experts with a specific competence in ethical issues.” | Dear Editor, we discuss the concern expressed in the recent revision and agree with your comment.  The quantitative and qualitative results of our research underline, in particular a general lack of information on ethics consultation service and activity carried out.  We believe that there is a need for further research to assess the characteristics and outcomes of an ethics consultation service.  Please, see the conclusion section revised accordingly to your comment. |
